# Supplementary material for: Ethnic disparities in COVID-19 mortality in Mexico: A cross-sectional study based on national data
Source: PLoS One. 2021 Mar 10;16(3):e0239168. doi: 10.1371/journal.pone.0239168 (PMC7946310; doi:10.1371/journal.pone.0239168)
Supplement: Attachment — Submitted filename: Response to Reviewers.docx [file pone.0239168.s002.docx]

**Response to Reviewers**

Reviewer 1 – Jennie Gamlin

**1. Greater clarity is needed about the use of terms race, ethnicity and indigenous. Indigenous is a category defined culturally and not biologically. It is not a category of race, but of social defined characteristics such as history and language. As the authors state, in the case of these data, indigeneity is defined as a speaker of indigenous language. In this case it would be good to a) state up front what the national definition is -are the national statistics on indigeneity also based solely on language? If not, can you state what the definition is (as opposed to the 'language' indicator in the COVID data)?**

Response: Dear Ms. Gamlin. First of all, thank you for your comments and questions. They have been of great help to us. Regarding this first comment, we agree that being Indigenous is defined culturally and not biologically. For this reason, we decided to add the three definitions in our manuscript (please see page 6-7, lines 127-133). We also added a new reference to support this (see reference Our analyses only included those who spoke an Indigenous language because that was the only variable available in the data set. Fortunately, this is the definition most commonly used in Mexico. We agree that being Indigenous is not a category of race, although Indigenous people suffer from a lot of racism in Mexico. We were more careful throughout the article with the use of these two words.

**2. In relation to the above, can you clarify the relationship between ethnicity and COVID mortality. For example, is language a barrier to care, are indigenous people less likely to seek care, are there different transmission patterns in indigenous localities and what are these.**

Response: Unfortunately, we were unable to determine if there are different transmission patterns in Indigenous localities because we had no access to this type of data. Furthermore, as far as we know, there are not available sources that report statistics specific to Indigenous communities and localities. Regarding access to care, one article did mention language barriers as a potential barrier. We added this to our discussion as we believe it is important to mention (see page 14, lines 240-243). We also discuss that only 1% of the total sample spoke an Indigenous language (compared to 6.5% of the population who report speaking an Indigenous language). This suggests an inequitable distribution of resources, testing and access to care (see page 14, lines 254-257). Thank you.

**3. What about Afro-Mexican populations. Are there any data on the incidence and morbidity of COVID among other non-indigenous ethnicities?**

Response: Thank you for this question. We believe it is an extremely important one. Unfortunately, we do not “measure” race the same way other countries do. We discuss why in page 15, lines 260-269. To this date, no studies have been done that attempt to compare skin color and COVID-19 outcomes.

**4. Consider if you actually need to use the term 'race' and whether this just muddies the water. There are no solid data (that I am aware of) that suggest a biological risk factor for specific racial characteristics, so race is really a proxy for social inequality. If this is the case, it should be stated and explained clearly.**

Response: Thank you for this suggestion. We chose to include the term race in our discussion because the literature uses both “race” and “ethnicity” as potential determinants of health in the COVID-19 pandemic. We understand that it is not being used in the literature to imply biological differences, but rather as a way to highlight social inequities, as you mention. In our article, we were careful with the use of the term “race”. In fact, we only mention it as a “self-identity” in the Mexican context in the discussion (see page 15, lines 262-266).

**5. What data are there on testing? So, for example, are indigenous people overrepresented in the mortality and hospitalisations data because they are testing less? Some context on this would help. So, for example with such a high rate of uninsured indigenous people and the excessive cost of testing in the private sector, the higher death rate could be due to the lower testing rate of indigenous people. Some mention of consideration of this is important to avoid this potential confounder.**

Response: Thank you for this question. The data set we used included all the people who had been tested up until August 3rd, 2020. As mentioned in lines 254-257, Only 4178 of our sample (COVID-19 positive) spoke an Indigenous language. We checked the total sample to include those who tested negative and only 9037 spoke an Indigenous language (around 1% of all those who were tested). This is considerably lower than the 6.5% of the Mexican population who speak an Indigenous language. We mention this is a limitation in page 15, lines 254-256 as well.

**6. In relation to 5), what is known about reporting of death rates and COVID in indigenous communities? Can you say anything about the localities where indigenous patients have died? Is there likely to be underrreporting?**

Response: Thank you. This is a great question. Unfortunately, no data on this is available in the literature or in official government sources. The data set we used includes “municipality of residence” as a variable. However, this does not mean that the person actually received attention there. The Mexican health system is using a sentinel surveillance system to diagnose COVID-19 cases, which means not every facility is actually testing suspicious patients. As mentioned above, we do believe there is underreporting because of the small proportion of Indigenous peoples being tested and diagnosed with COVID-19 (both around 1%).
