# Supplementary material for: Ethnic disparities in COVID-19 mortality in Mexico: A cross-sectional study based on national data
Source: PLoS One. 2021 Mar 10;16(3):e0239168. doi: 10.1371/journal.pone.0239168 (PMC7946310; doi:10.1371/journal.pone.0239168)
Supplement: Attachment — Submitted filename: Response to Reviewers.docx [file pone.0239168.s003.docx]

**Response to Reviewers**

**Editor**

**1. Please add in a 'limitation' section which picks up on the unanswerable questions (see original review comments as well) and considers the data in this light and comment on whether any new data has emerged since this paper was written which have any impact on the findings, since the situation is rapidly changing.**

**Reviewer**

**1. The authors have adequately responded to the concerns of the first review although, as they note in their response, some of the concerns cannot be addressed due to lack of data. For example, the extent of underreporting is not known, the low level of testing may skew results, it is not clear where in the country data correspond to. There are further concerns that the indigenous respondents only make up 1% of the dataset, which means that they are very considerably underrepresented in data. This being the case it would be helpful if the authors could add in a 'limitation' section which picks up on these unanswerable questions (see original review comments as well) and considers the data in this light. I also wonder if there are any new data which have emerged since this paper was written which have any impact on the findings, since the situation is rapidly changing it would be worth thinking about this.**

Response: Thank you both for your feedback and suggestions. We have made further changes to our limitations section in the Discussion. (See page 14, lines 248-252 and page 15, lines 263 and 267). Just an update regarding our testing strategy: it has not changed, unfortunately. The sentinel surveillance system is still in place, and the government has not announced any new plan regarding testing or focus on Indigenous communities. Furthermore, we thought it would be important to mention the lack of trust Indigenous communities have on the government, as this week an entire community of 40,000 Indigenous peoples are refusing to vaccinate because they do not trust the government.
